# Retroviral Integration Process in the Human Genome: Is It Really Non-Random? A New Statistical Approach

**DOI:** 10.1371/journal.pcbi.1000144

**Published:** 2008-08-08

**Authors:** Alessandro Ambrosi, Claudia Cattoglio, Clelia Di Serio

**Affiliations:** 1 University Centre for Statistics in the Biomedical Sciences, Università Vita-Salute San Raffaele, Milan Italy; 2 Italian Institute of Technology, Unit of Molecular Neuroscience, Istituto Scientifico H. San Raffaele, Milan, Italy; Washington University, United States of America

## Abstract

Retroviral vectors are widely used in gene therapy to introduce therapeutic genes into patients' cells, since, once delivered to the nucleus, the genes of interest are stably inserted (integrated) into the target cell genome. There is now compelling evidence that integration of retroviral vectors follows non-random patterns in mammalian genome, with a preference for active genes and regulatory regions. In particular, Moloney Leukemia Virus (MLV)–derived vectors show a tendency to integrate in the proximity of the transcription start site (TSS) of genes, occasionally resulting in the deregulation of gene expression and, where proto-oncogenes are targeted, in tumor initiation. This has drawn the attention of the scientific community to the molecular determinants of the retroviral integration process as well as to statistical methods to evaluate the genome-wide distribution of integration sites. In recent approaches, the observed distribution of MLV integration distances (IDs) from the TSS of the nearest gene is assumed to be non-random by empirical comparison with a random distribution generated by computational simulation procedures. To provide a statistical procedure to test the randomness of the retroviral insertion pattern, we propose a probability model (Beta distribution) based on IDs between two consecutive genes. We apply the procedure to a set of 595 unique MLV insertion sites retrieved from human hematopoietic stem/progenitor cells. The statistical goodness of fit test shows the suitability of this distribution to the observed data. Our statistical analysis confirms the preference of MLV-based vectors to integrate in promoter-proximal regions.

## Author Summary

Understanding how retroviral vectors (such as Moloney Leukemia Virus–based vectors) integrate in the human genome became a major safety issue in the field of gene therapy, since a concrete risk of developing tumors associated with the integration process was assessed in the clinical setting. Moloney Leukemia Virus–based vectors are apparently characterized by a non-random integration pattern, with a preference for the vicinities of active gene transcription start sites. We approach the problem of non-random retroviral integration from a probabilistic point of view. We model a normalized integration distance from the transcription start site of the nearest upstream or downstream gene. From this model, we derive a simple and straightforward testing procedure to estimate how the transcription start site of a given gene may or may not attract integration events. Our approach overcomes the issues of different gene length, gene orientation, and gene density, which are often critical in analyzing integration distances from transcription start sites. The approach is tested on real experimental data retrieved from human hematopoietic stem/progenitor cells.

## Introduction

The transfer of a therapeutic gene into somatic cells (gene therapy) is a promising medical approach for the management of many inherited and acquired diseases. Among several systems developed for gene delivery, replication-defective viral vectors derived from retroviruses are the most widely used. In fact, after infecting a target cell, retroviral vectors deliver the therapeutic gene directly to the cell nucleus and stably insert it into the host cell genome; the process is commonly referred to as “integration”.

It has been observed that retroviral vectors integrating in the proximity of the transcription start site (TSS) of host genes may enhance or disrupt normal transcription [1], occasionally favouring tumour initiation [2],[3] (insertional oncogenesis). Such genotoxic risk represents a major hurdle to the safety of gene therapy and requires sensitive pre-clinical assays for insertional mutagenesis [4],[5].

Understanding location preferences of retroviruses becomes crucial in evaluating both the safety profile of a therapeutic vector as well as the integration process *per se*, which is still far from being completely understood.

**Figure 1:**
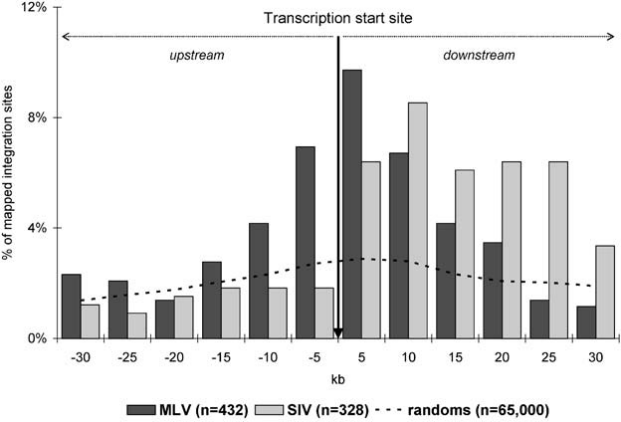
Distribution of Moloney Leukemia Virus (MLV) and Simian Immunodeficiency Virus (SIV) integration sites centered on transcription start sites of the nearest gene. The empirical comparison between simulated (dotted line) and observed distribution leads the authors to conclude in favour of non-randomness of retroviral integration.

Just few years ago, retrovirus integration was believed to be random, and the chance of accidentally activating a gene was considered remote. Recent studies based on cellular and animal models (reviewed in [6]) reported empirical evidence of preference for certain retroviral vectors, i.e. those deriving from Moloney Murine Leukemia Virus (MLV), to integrate near the start of transcriptional units, whereas others (like Simian Immunodeficiency Virus (SIV)– and Human Immunodeficiency Virus (HIV)–based vectors) did not show the same tendency. A representative example is given in Figure 1 (see [7]). In this case, the variable of interest to investigate integration preferences is the integration distance (ID) from the TSS of the nearest gene. In statistical terms, this is a signed distance function [8],[9], since it assumes negative or positive values according to the position of integration site with respect to the gene (upstream and downstream, respectively). The distribution of MLV IDs from the TSS shows a bell shape [10]. Here we remark that “bell-like” shape does not necessarily mean a “Gaussian” distribution. Indeed, other distributions (e.g., Cauchy distribution, Laplace distribution) may show a “bell-shape” similar to that observed in Figure 1. This is considered by the authors as sufficient evidence of a non-random pattern when compared to the almost flat distribution of 65,000 computer-generated random insertion sites. A crucial issue for mathematical biologists is to provide an analytic approach for the assessment of such non-randomness [11].

In this paper, we first show that a bell-shape distribution is not necessarily evidence of non-randomness. Then we introduce a new distance measure based on a normalization of the conventional ID. This new variable is assumed to follow a Beta distribution, thus allowing us to build a direct testing procedure for the non-random integration hypothesis. Applied to real experimental data, the estimated parameters provide a statistical measure confirming retroviral integration preferences for the proximity of TSSs.

## Methods

### Definitions

Each retroviral integration is defined by its nucleotide position on the chromosome (UCSC Genome Browser, human genome assembly March 2006, hg18 release, http://genome.ucsc.edu/). Integration-proximal genes are annotated according to UCSC RefSeq Genes category. For each insertion site (IS), the following definitions are uniquely given:

- nearest gene: nearest 3′ or 5′ end of a gene
- nearest upstream TSS
- nearest downstream TSS

**Figure 2:**
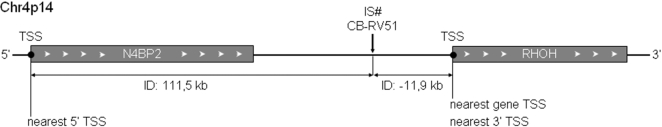
Example of integration distance calculation for one integration site mapped on Chromosome 4 (CB-RV51 insertion site in [20] dataset). Notice that in this particular case the transcription start site (TSS) of the nearest gene coincides with the nearest downstream (3′) TSS.

These definitions are applied to integrations landing within transcriptional units (intragenic) as well as to insertions mapping between two genes (intergenic). Integration distances from the nearest gene TSS and from the nearest 5′ and 3′ TSSs are then computed. IDs assume positive or negative values when the insertion nucleotide is located downstream or upstream of the TSS, respectively. Figure 2 provides a schematic representation of one intergenic integration from our dataset with the nearest transcriptional units. The IDs from the TSS relevant to this paper are shown.

### Modelling Integration Distance Distribution

Let *X* be the random variable (r.v.) describing the integration position. We next address the problem of testing the hypothesis of randomness of *X* over the genome with respect to the TSS. In statistical terms, this is equivalent to testing that the null hypothesis *H*_0_: *X* is distributed uniformly over the whole genome. The alternative hypothesis is *H*_1_: *X* distribution is influenced by the TSS.

Starting from a common annotation criteria [2],[7],[12],[13], we focus on ID from the TSS of the nearest 3′ or 5′ end of a gene (which might differ from the ID from the nearest TSS). We call this distance *Y*(*X*) defined as a function of *X*:
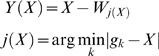
(1)where *W_j_*_(*X*)_ represents the TSS position of the nearest annotated gene *g_k_*.

**Figure 3:**
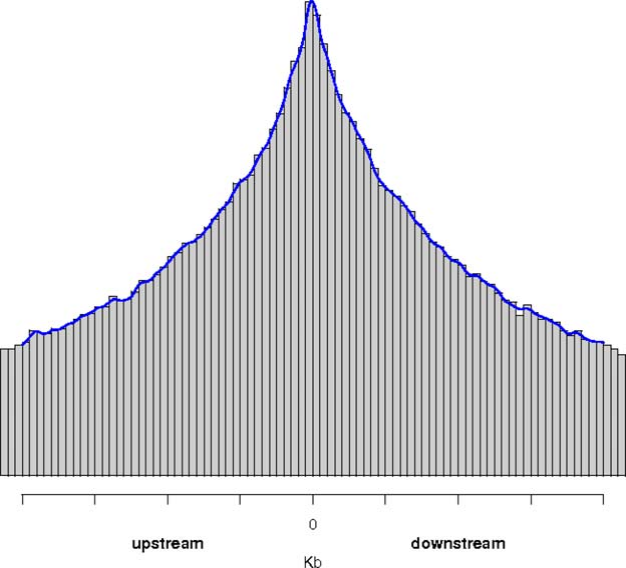
Distribution of 1,250,000 integration distances (kb) from the transcription start site (TSS) of the nearest gene (*Y*) randomly generated from a Uniform distribution. The solid line is the kernel density estimate plotted within a ±30 kb window for a better graphical visualization of the ”bell-shape” curve.

**Figure 4:**
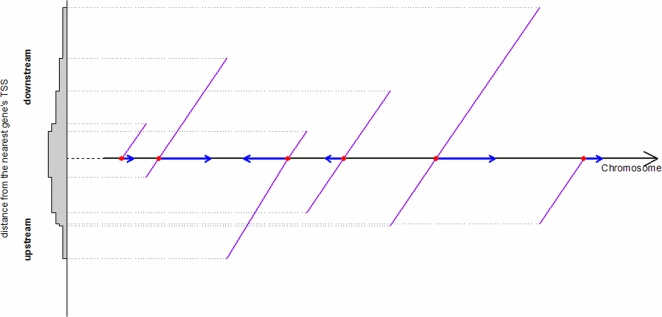
Integration distance (ID) from the nearest gene transcription start site (TSS). In this picture, six hypothetical genes with different length and orientation (blue arrows) are scattered along a chromosome (x-axis). The purple piecewise linear function represents the distance from the TSS of the nearest gene. This function has discontinuities exactly in the middle of the intervals between two consecutive genes. Even assuming a series of random integrations in this setting, we obtain a distribution of distances from TSSs (projected on the y-axis, gray plot) which is a mixture of Uniform distributions. As a consequence, the bell-shape curve is observed. Notice that the ID distribution is asymmetric around zero, since gene orientations and gene lengths determine which is the TSS to be considered in computing the distances (a symmetric distribution would be observed plotting the distance from the nearest TSS instead of the nearest gene TSS, data not shown).

Let us now suppose random integration, that is *X* is uniformly distributed over the genome. Despite this, it can be seen that *Y* might well be non-uniformly distributed. This is shown in Figure 3, where 1,250,000 integrations are generated from a Uniform distribution over the support [1, ∼3×10^9^ *bases*] and *Y*(*X*) are computed with respect to real TSSs and gene length distributions (Text S1, Remark 1). We can observe a bell-shaped distribution similar to that of MLV in Figure 1. This is not counter-intuitive given the uneven distribution of gene lengths and distances in the human genome. As a result, short IDs are more likely to be observed, whereas large IDs can only be observed for long genes and/or long intergenic distances; thus, they are less probable (see Figure 4). In fact, it can be proven that the exact distribution of *Y* is a mixture of Uniform distributions having support over the (signed) distances between two consecutive start sites. Thus, different gene lengths and gene orientations *per se* produce the bell-shaped ID distribution no matter what the integration preferences are.

We next build a new testing procedure for non-randomness. We start by normalizing the r.v. *Y*(*X*) (for simplicity hereafter denoted by *Y*). We define the IDs from the nearest downstream (*Y_D_*) and upstream (*Y_U_*) TSSs as:
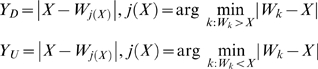
(2)


Let *Y* ^*^ be a new r.v. given by:
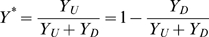
(3)which describes the ID as a proportion of the total distance between the start sites of two consecutive genes. Notice that *Y* ^*^ now becomes independent of *gene length*, *gene orientation*, and *gene density*, being always 0≤*Y* ^*^≤1. In statistical terms, we assume as a convenient distribution for *Y* ^*^ the Beta distribution, which is one of the most widely used in clinical, biological, and genetic settings (Bayesian frameworks [14],[15]). In fact, Beta distribution models events are constrained to take value within a finite interval (Text S1, Remark 2). This includes as a particular case the Uniform distribution on support [0,1], which coincides with our null hypothesis of random integration. For these reasons, the Beta distribution looks very suitable to describe, within the same parametric family, the integration preferences. This distribution family depends on two free parameters, *p* and *q*. The probability density function is given by:
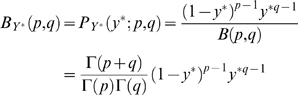
(4)with 0≤*Y* ^*^≤1 and 0 otherwise, *p*>0, *q*>0.

**Figure 5:**
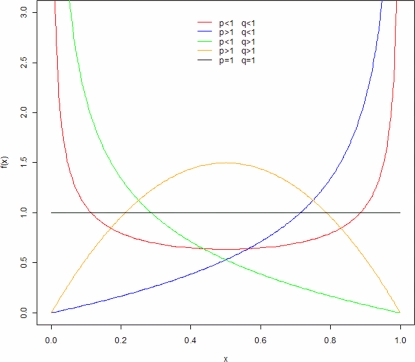
Beta probability distribution functions for different parameter combinations. Solid black line represents the case of Uniform distribution (*p* = *q* = 1). Other curves are all consistent with the alternative hypothesis in *H*_1_: *p*≠1 or *q*≠1.

The main aim of the modelling is the estimation of the parameters *p* and *q*. The null hypothesis “*X* is distributed uniformly over the whole genome” corresponds to “*Y* ^*^ is uniformly distributed in [0,1]”, that is equivalent to a Beta distribution with both *p* and *q* equal to one. The parameter estimates have also a practical interpretation: different values of *p* and *q* reflect different integration preferences as in Figure 5. This can also be easily visualized: a “U” shape in the distribution of *Y* ^*^ indicates that integrations land close to a TSS with higher probability (TSS *attracts* integrations). This occurs when both the beta parameters *p* and *q* are less than 1. On the contrary, *p* and *q* greater than 1 means that integration around a TSS is *disfavoured*. A straight line for *Y* ^*^ distribution (*p* = *q* = 1) indicates that integrations are randomly located with respect to a TSS.

In summary, we can now redefine the null hypothesis of random distribution of IDs in terms of values of the parameters (*p*,*q*), since the uniform distribution is a particular case of Beta, that is:
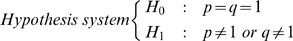
(5)To test the null hypothesis in Equation 5, we use Maximum Likelihood Estimators (MLEs; see Text S1, Remark 4) for the joint estimate of the parameters (*p*,*q*).

**Figure 6:**
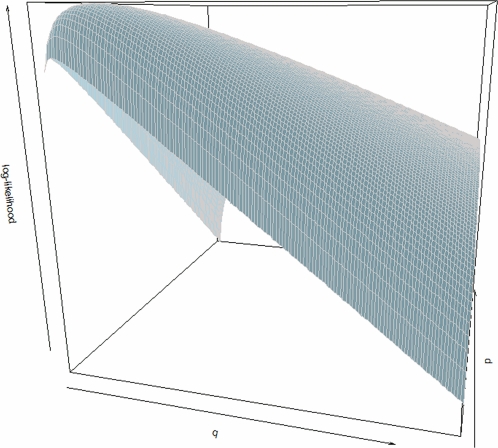
Loglikelihood function related to the distribution of *Y* ^*^ observed in human hematopoietic/stem progenitor cells showing the Maximum Likelihood Estimator (MLE) for the parameters *p* and *q*.

Method-of-Moments Estimates (MMEs) are also provided since it is well known that MMEs can be quickly and easily calculated (see Text S1, Remark 3), whereas the MLEs often involve more complex procedures (see Text S1, Remark 4). Typically, values for MLEs are obtained numerically by means of the Newton-Raphson method applied to the log-likelihood function (Figure 6). For more detailed comparison between the MMEs and MLEs for the parameters of a Beta (*p*,*q*) distribution, see [16],[17].

Comparison between observed and fitted IDs distribution to assess goodness of fit is performed by the Kolmogorov-Smirnov test. Confidence intervals of 95% are built on Bootstrap 50,000 replications [18]. We consider as an overall significance level *α* = 0.05.

Statistical analyses were performed with R-statistical software (ver. 2.6.1) [19].

## Results

We apply the testing procedure described in Equation 5 to a real experimental dataset. This includes 595 integrations retrieved from human hematopoietic stem/progenitor cells (CD34^+^ population) isolated from umbilical cord blood and infected in vitro with MLV-based retroviral vectors (RV and SIN-RV datasets in [20]). Integration analysis was performed 2 weeks after transduction, extracting genomic DNA from cells that underwent a maximum of 6 cell doublings (see [20] for more details about data and experimental procedures). The short-term culture period is a fundamental requirement to exclude a clonal selection effect, which indeed can occur in long-term culture or in vivo. This makes the dataset very suitable for investigating the integration preferences *per se* without confounding. The observed distribution of the ID from the TSS of the nearest genes is in accordance to the literature.

**Figure 7:**
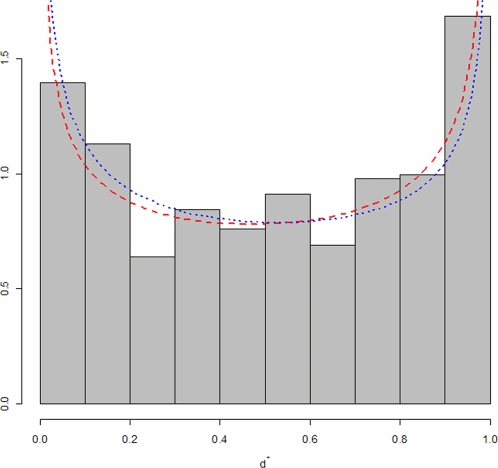
Comparison between the observed *Y* ^*^ distribution and the fitted distributions of Method of Moments Estimators (MMEs, red dashed line) and Maximum Likelihood Estimators (MLEs, blue dashed line). Goodness of fit was assessed by Kolmogorov Smirnov test (MME *p-value* = 0.909, MLE *p-value* = 0.8012).

In Figure 7, the observed distribution and fitted Beta distribution are plotted together. Goodness of fit for Beta distribution is assessed by Kolmogorov-Smirnov test (*p-value* = 0.8012). The “U” shape shown by a graphical investigation in Figure 7 suggests some evidence against random integration hypothesis. According to the hypothesis system, we estimate integration preferences by MMEs obtaining separate confidence intervals for *p̃* and *q̃* and by MLEs (*pˆ* and *qˆ*) to obtain *p-values* for the joint test in Equation 5. Estimation results are reported in Table 1. Parameter estimates are always less than 1 with an associated *p-value*<0.0001, leading to rejection of the hypothesis of uniformity of *Y* ^*^ in favour of the hypothesis that the TSS “attracts” integrations.

**Table 1:** Method of Moments and Maximum Likelihood *p* and *q* estimates (MME and MLE, respectively).

	MME (95% CI)	MLE	*p-value* for hypothesis system 5
*p*	0.568 (0.502–0.646)	0.599	<0.0001
*q*	0.551 (0.488–0.623)	0.592	<0.0001

## Discussion

Tumorigenesis induced by slow-transforming retroviruses occurs by insertional activation or deregulation of cellular proto-oncogenes by viral LTRs. Recent observations from gene therapy trials and pre-clinical models pointed out that MLV-derived retroviral vectors still retain this transforming ability, even if at a lower extent. Such genotoxic risk is augmented by MLV tendency to integrate near the TSS of host genes, where LTR transactivation can be more effective. For safety reasons, it becomes therefore crucial to understand the basis for retroviral integration site selection.

The goal of this paper is to provide a simple statistical tool to test whether integration data are distributed randomly over mammalian genome, in particular with respect to the transcription start site of genes surrounding integration events.

Our starting point is that integration distances generated in silico from a Uniform distribution show a bell-like shape as a consequence of different gene lengths and intergenic distances over the genome. Thus, when such shape is observed, it cannot automatically be interpreted as evidence of *non-random* integration distribution.

We propose a new method based on modelling the probability distribution function of IDs between two consecutive start sites. The normalized distance is assumed to follow a Beta distribution, both for statistical tractability and for suitability to the biomedical framework. This method differs from the commonly used simulation techniques to the extent that it models fully parametrically the ID distribution, with no need for a computationally demanding procedure. A big advantage of the proposed approach with respect to simulation procedures derives from the natural interpretation of Beta parameters. As seen in Figure 5, we can investigate how the TSS influences integration site selection: both “TSS attraction” (*p* and *q* less than 1) and “TSS repulsion” (*p* and *q* greater than 1) can now be tested. Notice that this information is not provided by the non-parametric Kolmogorov-Smirnov test for homogeneity of distributions, which verifies only whether two distributions are different but is not able to measure in which direction.

Estimation results derived from real experimental data show a U shape of the Beta distribution with a higher probability assigned to values in proximity of the TSS. Our statistical analysis confirms (also in human hematopoietic stem/progenitor cells) the preference of MLV-derived vectors to integrate in promoter-proximal regions, suggesting that the viral integrating machinery interacts preferentially with factors bound in the proximity of gene TSSs.

## Supporting Information

Text S1 Supplementary Material
(0.04 MB PDF)
