# Supplementary material for: Retroviral Integration Process in the Human Genome: Is It Really Non-Random? A New Statistical Approach
Source: PLoS Comput Biol. 2008 Aug 8;4(8):e1000144. doi: 10.1371/journal.pcbi.1000144 (PMC2453317; doi:10.1371/journal.pcbi.1000144)
Supplement: Text S1 — Supplementary Material (0.04 MB PDF) [file pcbi.1000144.s001.pdf]

Supplementary material

Remark 1. *Uniform distribution: the probability density function of the continuous uniform distribution is:*

$$f_X(x) = \begin{cases} \frac{1}{b-a} & \text{for } a \le x \le b, \\ 0 & \text{otherwise} \end{cases}$$

where a is also called location parameter and $(b-a)$ is the scale parameter.

Remark 2. *Beta distribution is based on the Beta function $B(p,q)$ defined for $p > 0$ and $q > 0$ by:*

$$B(p,q) = \int_{(0,1)} x^{p-1}(1-x)^{q-1}\,dx.$$

The general formula for the probability density function of the Beta distribution Beta (p,q,a,b) is

$$f_X(x \mid p,q,a,b) = \frac{(x-a)^{p+1}(b-x)^{q-1}}{B(p,q)(b-a)^{p+q-1}}$$

defined for $a \le X \le b$. When $a = 0$ and $b = 1$ we have the Beta distribution given in equation. Note that if X has Beta (p,q,a,b) distribution, then $X^* = \frac{X-a}{b-a}$ has Beta distribution given in eq. .The parameters are linked to the mean μ and variance σ^2 by the relationships:

- $\mu = \frac{p}{p+q}$;
- $\sigma^2 = \frac{pq}{(p+q)^2(p+q+1)}$.

Remark 3. *The method of moments is a method of estimation of population parameters by equating sample moments with unobservable population moments and then solving those equations for the quantities to be estimated. The moments of the standard Beta distribution are easy to express in terms of the Beta function as: $E(X^k) = B(p+k,q)/B(p,q)$. From the above equation the Method of Moments Estimators (MMEs) are obtained:*

$$\tilde{p} = \bar{x}\left(\frac{\bar{x}(1-\bar{x})}{s^2} - 1\right)$$

$$\tilde{q} = (1-\bar{x})\left(\frac{\bar{x}(1-\bar{x})}{s^2} - 1\right)$$

where $\bar{x}$ is the sample mean and s^2 is the sample variance.

Remark 4. *Assume that a random sample $x_1, \ldots x_n$ has been observed for a r.v. X with probability density distribution given by eq.. The log-likelihood function is (see figure):*

$$l(p,q \mid x) = \log L(p,q \mid x) = n(\log\Gamma(p+q) - \log(\Gamma(p)) - \log\Gamma(q)$$
$$+ (p+1)\sum_{i=1}^{n}\log x_i + (q-1)\sum_{i=1}^{n}\log(1-x_i).$$

where $\Gamma(\alpha)$ *is the Gamma function* $\Gamma(\alpha)=\int_0^\infty x^{\alpha-1}e^{-x}dx$*. Hence the ML estimators* $\hat{p}$ *and* $\hat{q}$ *are given by the system of equations:*

$$\begin{cases} \psi(\hat{p})-\psi(\hat{p}+\hat{q})=n^{-1}\sum_{i=1}^{n}\log x_i \\ \psi(\hat{q})-\psi(\hat{p}+\hat{q})=n^{-1}\sum_{i=1}^{n}\log(1-x_i) \end{cases}$$

where $\psi(\bullet)$ *is the digamma function:* $\psi(x)=\frac{\partial}{\partial x}\log\Gamma(x)$*. The solutions of the system is obtained by Newton-Raphson method. The ML estimators are asymptotically normal, unbiased and strongly consistent. The Information matrix, is*

$$\boldsymbol{I}(p,q)=n\begin{bmatrix} -\psi'(p+q)+\psi'(p) & -\psi'(p+q) \\ -\psi'(p+q) & -\psi'(p+q)+\psi'(q) \end{bmatrix}$$

From the inverse $\boldsymbol{I}^{-1}(p,q)$ *the asymptotic variances and covariance of ML estimates can be obtained.*
